# Increased Low- and High-Frequency Oscillatory Activity in the Prefrontal Cortex of Fibromyalgia Patients

**DOI:** 10.3389/fnhum.2016.00111

**Published:** 2016-03-14

**Authors:** Manyoel Lim, June Sic Kim, Dajung J. Kim, Chun Kee Chung

**Affiliations:** ^1^Neuroscience Research Institute, Seoul National University College of MedicineSeoul, South Korea; ^2^Department of Brain and Cognitive Sciences, Seoul National University College of Natural SciencesSeoul, South Korea; ^3^Department of Neurosurgery, Seoul National University HospitalSeoul, South Korea

**Keywords:** fibromyalgia, thalamocortical dysrhythmia, prefrontal cortex, gamma oscillation, pain, magnetoencephalography

## Abstract

Recent human neuroimaging studies have suggested that fibromyalgia (FM), a chronic widespread pain disorder, exhibits altered thalamic structure and function. Since the thalamus has extensive reciprocal connection with the cortex, structural and functional thalamic alterations in FM might be linked to aberrant thalamocortical oscillation. This study investigated the presence of abnormal brain rhythmicity in low- and high-frequency bands during resting state in patients with FM and their relationship to clinical pain symptom. Spontaneous magnetoencephalography (MEG) activity was recorded in 18 females with FM and 18 age- and sex-matched healthy control (HC) subjects. The most remarkable finding was that FM patients had general increases in theta, beta and gamma power along with a slowing of the dominant alpha peak. Increased spectral powers in the theta-band were primarily localized to the left dorsolateral prefrontal (DLPFC) and orbitofrontal cortex (OFC). Beta and gamma over-activation were localized to insular, primary motor and primary and secondary somatosensory (S2) cortices, as well as the DLPFC and OFC. Furthermore, enhanced high-frequency oscillatory activities in the DLPFC and OFC were associated with higher affective pain scores in patients with FM. Our results demonstrate that FM patients feature enhanced low- and high-frequency oscillatory activity in the brain areas related to cognitive and emotional modulation of pain. Increased low- and high-frequency activity of the prefrontal cortex may contribute to persistent perception of pain in FM. Therapeutic intervention based on manipulating neural oscillation to restore normal thalamocortical rhythmicity may be beneficial to pain relief in FM.

## Introduction

Fibromyalgia (FM) is a chronic pain disorder characterized by the widespread pain and tenderness, and which is often accompanied by affective and cognitive symptoms (Wolfe et al., 1990, 2010; Bartley et al., 2009; Schmidt-Wilcke and Clauw, 2011). Although the underlying cause of the symptoms of FM remains elusive, recent neuroimaging findings suggest that abnormally increased cortical excitability and dysfunctional endogenous pain modulation are important in maintenance of FM pain (Jensen et al., 2009; Mhalla et al., 2010; Woolf, 2011; de Tommaso et al., 2011; Dailey et al., 2013; Villamar et al., 2013; Kim D. J. et al., 2014; Loggia et al., 2014; López-Solà et al., 2014; Pujol et al., 2014; Choi et al., 2015; Foerster et al., 2015; Lim et al., 2015).

Of particular note, the thalamus is the region of the brain that mediates transmission of sensory and pain signals to the cortex. Single-photon-emission computed tomography studies reported reduced resting regional cerebral blood flow in the thalamus in FM patients compared with healthy control (HC) subjects (Mountz et al., 1995; Kwiatek et al., 2000). Abnormal thalamic activity in FM was also reported in functional magnetic resonance imaging (fMRI) studies during a painful pressure stimulus. Patients with FM showed no thalamic increases (Gracely et al., 2002) or deactivation of the thalamus as opposed to increased activity in HC subjects (Jensen et al., 2009). A recent study reported the reduced functional connectivity between the thalamus and the lateral orbitofrontal cortex (OFC) in FM patients (Jensen et al., 2012b). FM has been also associated with altered brain structure in the thalamus in the form of decreased gray matter in the left posterior thalamus (Schmidt-Wilcke et al., 2007) and lower fractional anisotropy in the bilateral posterior thalami (Lutz et al., 2008). Since the thalamus has extensive reciprocal connection with the cortex (Jones, 2001), it seems likely that structural and functional thalamic alterations in FM might be linked to aberrant thalamocortical oscillation.

Thalamocortical dysrhythmia (TCD) has been proposed as the underlying mechanism of chronic neuropathic pain and other disorders (Llinás et al., 1999, 2005; Schulman et al., 2005, 2011). TCD in chronic pain is characterized by abnormal oscillatory activity in the form of increased spectral power and a dominant peak shifted towards a lower frequency (Llinás et al., 2005; Sarnthein et al., 2006; Stern et al., 2006; Jensen et al., 2013; Vuckovic et al., 2014). Spontaneous rhythmic activity measured by magnetoencephalography (MEG) or electroencephalography (EEG) shows enhanced theta and beta activity in patients with neuropathic pain compared to HCs (Sarnthein et al., 2006; Stern et al., 2006; Walton et al., 2010). Theta and beta bands over-activations in patients with neuropathic pain are localized to multiple area of the cortical pain matrix, including the prefrontal, anterior cingulate, and insular cortices as well as primary (S1) and secondary somatosensory (S2) cortices (Stern et al., 2006). These over-activations in the cortical pain matrix can be reduced after a therapeutic lesion in the central lateral nucleus of the thalamus (Stern et al., 2006). Therefore, TCD may perpetuate and exacerbate the chronic pain condition when abnormal rhythmic activity is present in the cortical pain network. Especially, high-frequency over-activation, referred to as an edge effect, can lead to pain symptoms (Llinás et al., 1999, 2005; Walton et al., 2010). However, to our knowledge, an abnormality in spontaneous brain activity in patients with FM has not been investigated.

The present study aimed to investigate the presence of abnormal brain rhythmicity in low- and high-frequency bands during resting state in patients with FM. By applying our MEG source localization approach to the cortical surface model (Kim and Chung, 2008; Kim J. S. et al., 2014), we further aimed to identify the cortical sources that generate different oscillatory activity between FM patients and HC subjects. We hypothesized that FM patients have increased spontaneous brain activity in both low- and high-frequency bands, and that these over-activations are related to clinical pain severity in FM patients.

## Patients and Methods

### Participants

The study participants were recruited from the outpatient clinics of the Rheumatology Departments of the Seoul National University Hospital and Hallym University Sacred Heart Hospital. Eligibility criteria for patients with FM were: (1) meeting the American College of Rheumatology 1990 criteria for primary FM (Wolfe et al., 1990); (2) a duration of widespread pain of at least 3 months but less than 10 years; (3) experiencing pain intensity of at least 40 on a 0–100 mm pain visual analog scale (VAS) over the past week; (4) age of 30–60 years; (5) female; (6) right-handed (Oldfield, 1971); and (7) willing to stop taking medications that might affect brain electrophysiology, such as analgesics, antidepressants, and anticonvulsants, at least 3 days before the assessments. Patients were excluded if they had: (1) secondary FM associated with inflammatory arthritis; (2) history of substance abuse; (3) signs of peripheral neuropathy or concomitant acute pain in the upper extremities; (4) hearing loss or use of hearing aids; (5) pregnancy or breastfeeding; or (6) contradictions for MEG or MRI assessments. Age-, gender-, and education-matched HC subjects were recruited by local advertisement. Exclusion criteria were the same as for patients. The study protocol was approved by the Institutional Review Boards at Seoul National University Hospital and Hallym University Sacred Heart Hospital and was conducted in compliance with the Declaration of Helsinki. All participants (19 FM patients and 21 HC subjects) provided written informed consent.

The Beck Depression Inventory (BDI; Beck et al., 1961), and Beck Anxiety Inventory (BAI; Beck et al., 1988) were assessed. The Fibromyalgia Impact Questionnaire (FIQ; Burckhardt et al., 1991) was used to assess the composite impact of FM symptoms. The sensory and affective components of pain were assessed using the short-form McGill Pain Questionnaire (SF-MPQ; Melzack, 1987). The questionnaires were performed on the same day as the MEG recordings, except for nine subjects. For nine subjects, the questionnaires were completed at the first visit and resting MEG data was acquired at the second visit. The mean ± standard deviation (SD) interval between sessions was 12.3 ± 5.7 days for FM (*n* = 6) and 18.3 ± 4.2 days for HC subjects (*n* = 3). The demographic and clinical characteristics of the participants are presented in Table 1.

**Table 1:** **Demographic and clinical characteristics of study participants**.

	FM (*n* = 18)	HC (*n* = 18)	*P* value
Age, *year*	45.1 ± 8.5	44.7 ± 8.8	0.89
Education, *year*	13.1 ± 2.3	13.3 ± 2.4	0.83
Duration of illness, *month*	36.6 ± 31.7	NA	NA
Medication, n (%)
Analgesics/muscle relaxants/NSAIDs	12 (67%)	NA	NA
Antidepressants	13 (72%)	NA	NA
Anticonvulsants	6 (33%)	NA	NA
Beck Depression Index score	19.0 ± 7.0	3.3 ± 4.1	<0.001
Beck Anxiety Index score	23.8 ± 10.9	1.8 ± 2.1	<0.001
Fibromyalgia Impact Questionnaire	62.0 ± 13.4	NA	NA
SF-MPQ (sensory)	14.4 ± 6.8	NA	NA
SF-MPQ (affective)	5.9 ± 2.6	NA	NA
SF-MPQ (total)	20.3 ± 9.0	NA	NA

*Data are presented as mean ± standard deviation or number of subjects (%). FM, fibromyalgia; HC, healthy control; NSAIDs, nonsteroidal anti-inflammatory drugs; SF-MPQ, short-form McGill Pain Questionnaire; NA, not applicable*.

### Experimental Conditions

Subjects were asked to refrain from consuming caffeinated beverages before the recording to avoid the caffeine-induced theta power decrease (Landolt et al., 2004). Spontaneous brain activity was consecutively recorded with subjects in a resting state with their eyes-open and resting state with their eyes-closed. In the eyes-open condition, the subjects were instructed to keep their gaze fixed on a cross in the center of the front wall. Subjects were instructed to relax, but to stay alert during the recording. Subject’s alertness during the experiment was checked by self-report after the MEG recording. The recording time of each condition was 270 s. The experimenter (M.L) monitored the subject’s head online using a video camera throughout the recordings. All subjects were carefully immobilized and did not markedly move their heads during the recordings. We focused our analysis on the eyes-closed condition (Sarnthein et al., 2006), as this condition is a relatively straightforward means to standardize the experiment between healthy and patient groups, and can minimize eye movements, such as eye blinks, which can affect frontal brain activity (van Diessen et al., 2015).

### MEG Acquisition

MEG and MRI data were acquired at Seoul National University Hospital. Spontaneous MEG signals were recorded with a VectorView^TM^ 306-channel whole-head neuromagnetometer (Elekta Neuromag, Helsinki, Finland) in a magnetically shielded room. The device contains 102 identical triple sensors, comprising two orthogonal planar gradiometers and one magnetometer. During the MEG recordings, the subjects sat comfortably under the helmet-shaped sensor array and were asked to keep their heads as still as possible. The exact head position with respect to the sensors was determined by measuring the magnetic signals produced by currents delivered to four head position indicator coils placed at known sites on the scalp. The two groups did not differ in their head position relative to the MEG sensors. The location of head position indicator coils and three anatomical landmarks, the nasion and two preauricular points, were measured by using a FASTRAK^TM^ three-dimensional digitizer (Polhemus, Colchester, VT, USA), to allow alignment of the MEG and MRI coordinate systems. The *x*-axis passed through the two preauricular points with the positive direction to the right. The positive *y*-axis passed through the nasion, and the *z*-axis pointed upward. The MEG signals were band-pass filtered at 0.1–300 Hz and sampled at 1 kHz. To reduce environmental and biological noise, we applied the spatiotemporal signal space separation method using MaxFilter software version 2.2.10 (Elekta Neuromag; Taulu and Simola, 2006; Lim et al., 2014, 2015; Choi et al., 2015).

### MRI Acquisition

T1-weighted brain MRI was acquired for each subject using a Magnetom TrioTim 3T scanner (Siemens, Erlangen, Germany). Parameters were: sagittal acquisition with a 256 × 256 matrix; field of view = 250 mm; voxel size = 1 mm × 1 mm × 1 mm; slice thickness = 1.0 mm with no gap; repetition time/echo time = 1670/1.89 ms; flip angle = 9°; 1 excitation. One HC subject whose MRI examination could not be completed due to claustrophobia was excluded from further analysis. We found no group differences in whole-brain volume measures in our previous study (Kim D. J. et al., 2014).

### Source Imaging

We applied the constrained Laplacian anatomic segmentation using a proximity algorithm to reconstruct the inner and outer interfaces of the gray matter with 40,962 vertices for each hemisphere (Kim et al., 2005). A mid-surface was generated by averaging the corresponding points of the inner and outer interfaces. The vertices consisting of the mid surface were down-sampled to 5 mm intervals between points. The number of source points was about 8200 in the whole-brain model after the down-sampling. The source images were reconstructed on the down-sampled points. A linear interpolation was applied to display the source imaging on the cortical surface.

The solution of the forward problem has a linear relationship between the current sources and the measured signals:

B=A·S+n

where *B* is the vector of the measured signals, *S* is the vector of current sources, *A* is the lead field matrix, which describes the sensitivity pattern of each MEG sensor (magnetometers and gradiometers), and *n* is a noise vector. A spherical head model was used for source modeling (Hämäläinen et al., 1993). The center of the sphere was fit from manually selected inner skull points.

We used a standardized low-resolution brain electromagnetic tomography (sLORETA) algorithm (Pascual-Marqui, 2002) to localize the sources of oscillatory activity in various frequency bands: theta (5–8 Hz), alpha (8–13 Hz), beta (13–30 Hz), and gamma (30–48 Hz). The modified pseudo-statistics of sLORETA were used to analyze the absolute activation at each point (Kim and Chung, 2008; Kim J. S. et al., 2014). The weight matrix at the *j*-th point, *W_j_*, is given by:

$$W_{j}=\frac{{\left[A^{T}{\left(AA^{T}+\alpha I\right)}^{+}\right]}_{j}}{\sqrt{{\left[A^{T}{\left(AA^{T}+\alpha I\right)}^{+}A\right]}_{jj}}}$$

where *W_j_* is a row vector, *I* is the identity matrix, and *α* is the regularization parameter. For any matrix *M*, *M^+^* denotes its Moore-Penrose pseudo-inverse (Rao and Mitra, 1973). The denominator indicates the SD of the estimated current density.

The current density at the *j*-th point, *S_j_*, is given by:

$$S_{j}=W_{j}\cdot B$$

The signal at each polygonal point consisted of two orthogonal source vectors tangential to the surface of the spherical head model. Since two source activities are mutually orthogonal, source power at each location can be determined by vector addition.

### MEG Analysis

The MEG signals were segmented into 4 s epochs without overlap. Epochs with amplitudes exceeding 3000 fT/cm for MEG channel or 150 μV for electrooculogram were excluded from the average. The data of one FM and two HC subjects were excluded from further analyses because MEG signals were heavily contaminated by MEG sensor noise or eye movements. The mean ± SD artifact free epochs were 61.4 ± 1.5 and 60.8 ± 0.7 (FM and HC, respectively). All artifact free epochs were extracted through a Hanning window. Power spectral analysis was performed with a fast Fourier transform of 4096 points. The real and imaginary parts of fast Fourier transform at each source point were estimated separately using the spatial filter matrix described in the previous section of this article. The details of the methods were described in previous studies (Jensen and Vanni, 2002; Kim and Chung, 2008; Kim J. S. et al., 2014). The Fourier transformed signals *S^k^*(*f*) were calculated for each segment, *k*, with respect to the frequency of *f*. The real *S^k^*(*f)_Re_* and the imaginary part *S^k^*(*f)_Im_* of the Fourier-transformed signal were then applied to the sLORETA filter resulting in the current distributions, *Q^k^*(*f)_Re_* and *Q^k^*(*f)_Im_*, respectively. The absolute current estimates at the *j*-th source point for all the segments were averaged in the source space as follows:

$$Q_{j}(f)=\frac{1}{2M}\sum_{k=1}^{M}\|Q_{j}^{k}{\left(f\right)}_{Re}\|+\|Q_{j}^{k}{\left(f\right)}_{Im}\|$$

Finally, we divided the frequency spectrum into four frequency bands.

### Statistical Analyses

Individual surface models were nonlinearly transformed to the template using a surface registration algorithm (Robbins et al., 2004). The spatially normalized sLORETA images of the two groups were compared at each vertex using general linear models adjusting for age. We performed a permutation tests with a *P* value of 0.01 to determine whether significant differences were not due to chance in multiple comparisons (Bullmore et al., 1999). Subjects were randomly assigned to groups across 10,000 new randomized analyses at each vertex, and the number of significant results (i.e., the power spectral density at any vertex that differed significantly between groups at a threshold of *P* < 0.01) that occurred in the real test for group differences were compared with the null distribution of significant results that occurred by chance (Kim J. S. et al., 2014).

The regional power spectra were extracted from the locations showing the maximal difference of theta power in the dorsolateral prefrontal (DLPFC) and OFC areas. Relationships between clinical pain symptoms of FM patients and the spectral power in the high-frequency (beta and gamma) oscillations were tested using Pearson’s correlation coefficient. *P* values < 0.0125 were considered to indicate significance after Bonferroni correction for multiple comparisons. All group data are presented as mean ± SD.

## Results

### Power Spectral Analysis

**Figure 1:**
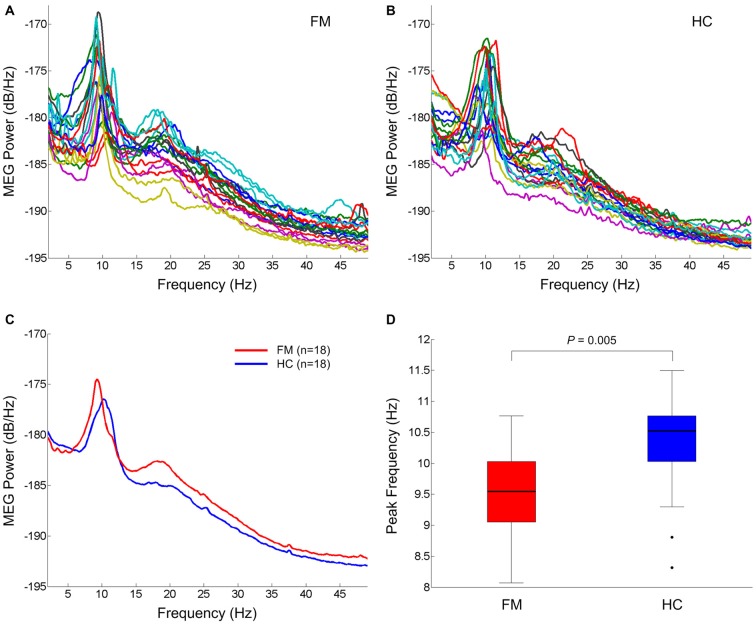
**Resting state oscillatory brain activity.** Superposition of individual mean power spectra of spontaneous brain activity recorded from all MEG gradiometer sensor pairs in FM patients **(A)** and HC subjects **(B)**. Grand averaged power spectra of FM patients (red) and HC subjects (blue) **(C)**. Box plots of the dominant peak frequency in the alpha band **(D)**. On each box, the horizontal line is the median and the edges of the box are the 25th and 75th percentiles. Whiskers extend to the highest and lowest values within 1.5 times the interquartile range. The filled circles represent outliers. *P* value was determined by the two-tailed *t*-test for independent samples. FM, fibromyalgia; HC, healthy control.

The individual global power spectra for the FM patients and HC subjects are superimposed in Figures 1A,B, respectively. Figure 1C shows the grand average power spectra in the resting state of the FM patients and HC subjects. Notably, FM patients had general increases in theta, beta and gamma power and a dominant alpha peak shifted towards a lower frequency. The mean dominant alpha peak of the FM group (9.6 ± 0.6 Hz) was lower than that of the HC group (10.3 ± 0.8 Hz; *P* = 0.005; Figure 1D).

### Source Localization

**Figure 2:**
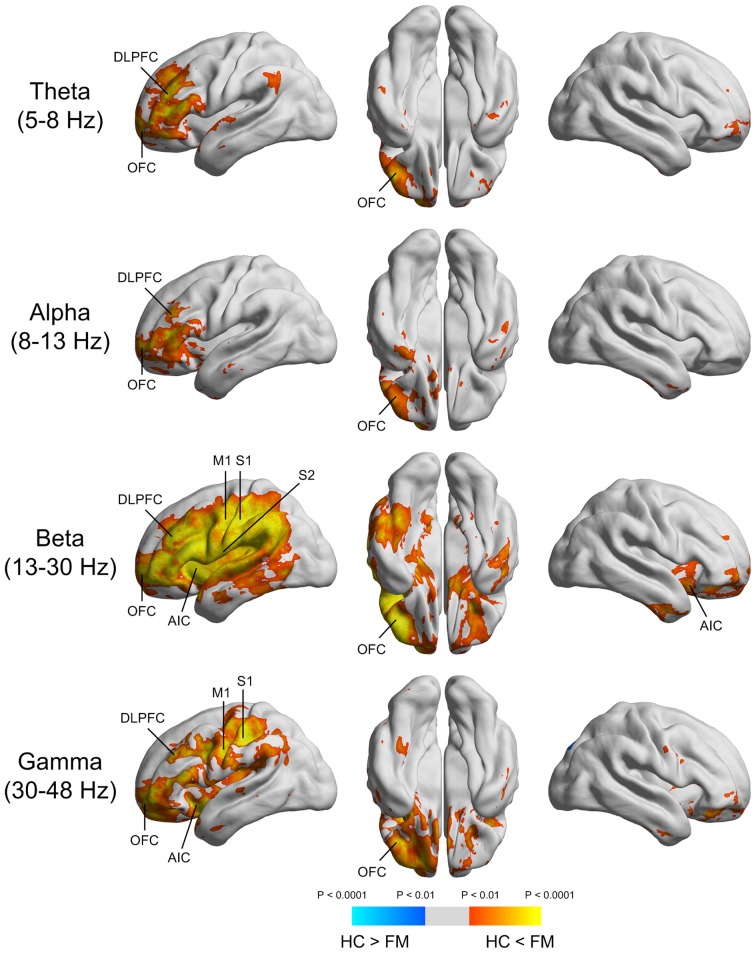
**Source imaging of the significant power differences in various frequency bands between FM patients and HC subjects.** DLPFC, dorsolateral prefrontal cortex; OFC, orbitofrontal cortex; AIC, anterior insular cortex; M1, primary motor cortex; S1, primary somatosensory cortex; S2, secondary somatosensory cortex.

Spontaneous peak over-activations within the theta (5–8 Hz), alpha (8–13 Hz), beta (13–30 Hz), and gamma (30–48 Hz) frequency bands were commonly localized to the left DLPFC and OFC (Figure 2). In the beta frequency band, peak over-activations were found in the anterior insular cortex (AIC) and primary motor cortex (M1), as well as S1 and S2 in the left hemisphere. Peak over-activations in the left AIC, M1, and S1 were also persistent in the gamma frequency band.

### Clinical Symptom Correlation

**Figure 3:**
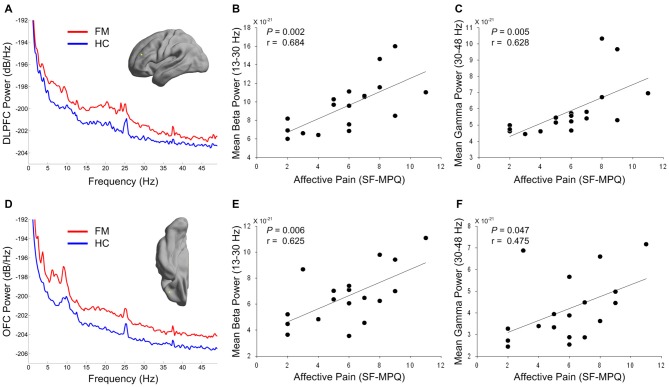
**Power spectrums of the prefrontal source activity (A,D) and relationship between affective pain intensity and high-frequency power of prefrontal regions in patients with FM (B,C,E,F).** The yellow dots in the cortical surface indicate the locations of the maximal difference in theta power in the DLPFC **(A)** and OFC **(D)** areas. Red and blue lines indicate grand averaged source power spectra for FM patients and HC subjects, respectively. Mean beta **(B,E)** and gamma **(C,F)** powers were calculated from the individual DLPFC **(A)** and OFC **(D)** source power spectra in FM patients. DLPFC, dorsolateral prefrontal cortex; OFC, orbitofrontal cortex; SF-MPQ, short-form McGill Pain Questionnaire; FM, fibromyalgia; HC, healthy control.

Figures 3A,D depict the power spectrum of the left DLPFC and OFC source, respectively. These areas are involved in cognitive-affective modulation of pain. Interestingly, abnormally increased rhythmicity was seen at not only low frequency, but also high frequency. Thus, we tested the relationship between over-activation in the high-frequency band in these areas and affective pain intensity in patients with FM. Both mean beta and gamma power in the DLPFC was positively associated with clinical ratings in the affective dimension of pain (*r* = 0.684, *P* = 0.002; *r* = 0.628, *P* = 0.005; Figures 3B,C). In addition, higher mean beta power in the OFC was also associated with higher score in the affective dimension of pain (*r* = 0.625, *P* = 0.006; Figure 3E). After correcting for multiple comparisons, higher mean gamma power in the OFC was marginally associated with increased affective pain intensity (*r* = 0.475, *P* = 0.047; Figure 3F). In our exploratory analysis, we did not observe any significant correlation between theta and alpha powers in the DLPFC or OFC and affective pain score in patients with FM (all *P*s > 0.05).

## Discussion

To our knowledge, this is the first study to investigate changes of spontaneous neuromagnetic activity during the resting state and their relationship with clinical pain symptoms in patients with FM. FM patients displayed general increases in theta, beta and gamma power, along with a slowing of the dominant alpha peak. Spontaneous oscillatory over-activations were generated primarily in the prefrontal, insular, S1, S2, and M1 areas. Increased high-frequency oscillatory activity in the DLPFC and OFC was associated with higher affective pain scores in patients with FM.

Our findings of abnormally increased low-frequency activity in FM patients are partly in agreement with TCD as a pathophysiological model for chronic neuropathic pain (Llinás et al., 1999, 2005; Schulman et al., 2005; Saab, 2012; Leblanc et al., 2014). A prior resting EEG study showed that neuropathic pain patients exhibited higher spectral power over the frequency range of 2–25 Hz with a dominant peak shifted towards a lower frequency (Sarnthein et al., 2006). The authors also reported reduced pain symptom accompanied by decreases in theta EEG power after therapeutic lesion in the thalamus (central lateral thalamotomy) in a subgroup of patients. This result suggests that both neuropathic pain and low-frequency oscillations are presumably determined by thalamocortical loops (Sarnthein et al., 2006). This notion is supported by the evidence of high theta coherence between EEG and thalamic local field potentials from central lateral nucleus in neuropathic pain patients (Sarnthein and Jeanmonod, 2008). In addition, a recent resting MEG study also revealed a leftward shift of the dominant peak in the power spectrum and higher mean spectral power ratio (4–8 Hz/8–12 Hz) in patients with complex regional pain syndrome (Walton et al., 2010). Therefore, although the resting state MEG activity does not directly reflect thalamus activity, our results indicate that the pathophysiology of FM may share some characteristics with TCD.

The most obvious characteristic is the enhanced spontaneous oscillatory activity in not only the theta but also the beta and gamma bands of FM patients compared to HC subjects. In the framework of TCD (Llinás et al., 1999, 2005), the deafferentation of excitatory inputs on thalamic cells results in cell membrane hyperpolarization. This in turn generates low-threshold calcium spike bursts at theta frequency by deinactivation of calcium T-channels (Llinás and Jahnsen, 1982). Low-threshold calcium spike bursts of thalamic relay neurons exert an oscillatory activity in the thalamocortical loops (Sarnthein and Jeanmonod, 2008) and lead to over-production of theta oscillatory activity in the cortex (Sarnthein et al., 2006; Stern et al., 2006; Walton et al., 2010). Since the DLPFC and OFC receive projections from the mediodorsal nucleus of the thalamus (Klein et al., 2010), theta frequency activity in the mediodorsal thalamus could lead to theta over-production in the DLFPC and OFC. In the final step, thalamocortical modules in theta mode disrupt lateral inhibition on other cortical modules and these modules are over-activated at high frequencies (beta and gamma), which constitutes the edge effect (Llinás et al., 1999, 2005). This edge effect has been suggested to be responsible for the positive symptoms of chronic pain patients by acting in the cortical pain matrix (Schulman et al., 2005; Stern et al., 2006; Walton et al., 2010).

In this study, both low- and high-frequency over-activations in FM patients were commonly generated in the DLPFC and OFC regions. Our results are partly consistent with a recent resting state functional MRI study showing that FM patients had increased spectral power at 0.01–0.08 Hz, which was thought to be related to brain activity fluctuation, in several brain regions associated with pain processing including the DLPFC (Kim et al., 2013). DLPFC activity has been implicated in the cognitive and attentional processing of pain (Lorenz et al., 2003; Wiech et al., 2008). In addition, neuronal gamma oscillation has been suggested to be involved in attentional effects of pain (Hauck et al., 2007; Tiemann et al., 2010). Thus, we reasoned that over-activation of high frequency power, including the beta and gamma bands, in the DLPFC during the resting state may at least reflect sustained attention to spontaneous pain in patients with FM. The present results of a positive relationship between affective pain symptom and high-frequency power in the DLPFC support this interpretation.

Since the OFC is directly connected to the limbic system including amygdala, it is critically involved in regulation of emotion (Kringelbach and Rolls, 2004; Kringelbach, 2005). There is interesting evidence that activation of the OFC is related to the affective aspects of touch; e.g., pleasant and painful touch, while the S1 is activated more by neutral touch (Rolls et al., 2003). A prior study in patients with peripheral nerve injury revealed that brush-evoked allodynia activated the OFC and AIC, possibly due to the stronger emotional load of neuropathic pain (Witting et al., 2006). Based on these findings, we speculate that abnormally increased high-frequency power in the OFC may indicate emotional and affective dysregulation during resting state in FM. This hyperexcitation state would result in an overall decrease in brain resources for processing in emotional modulation. In this context, a recent study reported that a deficit in modulation of painful stimuli in a positive emotional context is related to less brain activation, including in the OFC, for FM patients (Kamping et al., 2013). FM has been also associated with structural change; i.e., increase in gray matter in the left OFC (Schmidt-Wilcke et al., 2007). Thus, the observed increases in spectral power in the OFC could be a consequence of its increased gray matter volume. Given a finite degree of synchronous firing, a higher cell population would lead to a stronger signal. Taken together, the functional and structural abnormalities of the OFC may contribute to altered emotional regulation of pain in FM (González-Roldán et al., 2013; Fallon et al., 2015).

The results of beta and gamma over-activation in the S1, S2 and M1 further support the view of altered excitability of the sensorimotor system in patients with FM (Mhalla et al., 2010, 2011; Lim et al., 2015). Recent studies suggested that beta and gamma oscillatory activity in the sensorimotor cortex have an important role in encoding of pain. Previous studies showed that painful stimuli suppress spontaneous beta-band oscillation in the sensorimotor cortex (Ploner et al., 2006; Kirveskari et al., 2010). In addition, pain induced gamma-band activity in the S1 (Gross et al., 2007; Hauck et al., 2013) and sensorimotor (Hauck et al., 2007) cortex was positively associated with the subjective perception of pain. Therefore, further studies are needed to investigate how the neuronal oscillatory response to painful stimuli in the sensorimotor cortex influence changes in the perception and processing of pain in FM. For example, a recent EEG study that reported beta-band suppression in the ipsilateral S1 and S2 regions during mechanical brush-evoked allodynia in FM patients indicated altered oscillatory processing of innocuous somatosensory input, which may then affect clinical symptom severity (Fallon et al., 2013).

Recent studies using multiple brain imaging modalities have demonstrated that chronic neuropathic pain is associated with structural and functional thalamic alterations, which may result in development of TCD (Henderson et al., 2013; Gustin et al., 2014; Youssef et al., 2014). Specifically, patients with painful trigeminal neuropathy display a reduction in gray matter volume in the somatosensory thalamus (ventroposterior nucleus) and reduced blood flow in the thalamic reticular nucleus (Henderson et al., 2013). The findings suggested that reduction of thalamic GABA level, likely as a consequence of decreased thalamic reticular nucleus activity, leads to altered thalamocortical rhythmic oscillation, which in turn results in the perception of persistent pain (Henderson et al., 2013). Therefore, we reasoned that abnormal spontaneous oscillatory activity in FM patients may be due to structural (Schmidt-Wilcke et al., 2007; Lutz et al., 2008) and functional (Mountz et al., 1995; Kwiatek et al., 2000; Gracely et al., 2002; Jensen et al., 2009, 2012a,b; Garcia-Larrea and Peyron, 2013) changes in the thalamus.

Several limitations should be considered in interpreting our results. First, since we did not measure thalamic activity, the present study could not provide direct evidence of thalamic dysregulation of cortical activity in FM. Second, although patients stopped all medications 3 days before the MEG recording, this may not have been sufficient time to exclude the long-term effect of medication on cortical excitability. On the other hand, this medication-discontinuation could have led to withdrawal symptoms. We confirmed that every patient was in good condition before and after the experiments and related problem was not reported by every patient. Even if the patients had symptoms from the discontinuation, these may have been minimal or not to the extent that could be clearly differentiated. Third, our source imaging results were lateralized to the left hemisphere. We have no clear explanation for this finding. One possibility is that abnormal thalamocortical low frequency activity triggered in the left prefrontal regions may cause less collateral inhibition on neighboring cortical modules, thereby promoting over-production of high frequency activity (Llinás et al., 1999). Further analysis of intra- and inter-hemispheric cross-frequency coupling (Adamchic et al., 2014; De Ridder et al., 2015) would facilitate the characterization of pathological oscillatory dynamics across brain networks in FM. Lastly, the source level findings would benefit from a more advanced forward and inverse modeling approach. In this study, our use of the spherical head model is a kind of compromise between precision and practicability. However, the oversimplified single-sphere model might have resulted in localization errors and better accuracy could have been obtained with a more realistic head model, especially for frontal areas (Stenroos et al., 2014). This means that there is some uncertainty whether the activity really originates from DLPFC and OFC. There is a pressing need for future studies which use a more realistic head model in order to confirm the validity of our results. In addition, although sLORETA could result in robust source regions, the source image can be blurred. The use of beamforming techniques could help identify frequency band-specific neural sources more precisely (Hillebrand et al., 2005).

In summary, this study demonstrates that FM patients present enhanced low- and high-frequency oscillatory activity in the brain areas related to cognitive and emotional modulation of pain. Increased low- and high-frequency activity of the prefrontal cortex may contribute to persistent perception of pain in FM. Therapeutic intervention based on manipulating neural oscillation to restore normal thalamocortical rhythmicity may be beneficial to pain relief in FM.

## Author Contributions

All authors were involved in revising the article critically for important intellectual content. CKC had full access to all of the data in the study and takes responsibility for the integrity of the data and accuracy of the data analysis. ML, JSK, DJK, and CKC: study conception and design, acquisition of data, and analysis and interpretation of data. ML: drafting the manuscript.

## Conflict of Interest Statement

The authors declare that the research was conducted in the absence of any commercial or financial relationships that could be construed as a potential conflict of interest.
